# Peanut butter feeding induces oral tolerance in genetically diverse collaborative cross mice

**DOI:** 10.3389/falgy.2023.1219268

**Published:** 2023-07-17

**Authors:** Robert M. Immormino, Johanna M. Smeekens, Priscilla I. Mathai, Janelle R. Kesselring, Andrew V. Turner, Michael D. Kulis, Timothy P. Moran

**Affiliations:** ^1^ Department of Pediatrics, UNC School of Medicine, Chapel Hill, NC, United States; ^2^ UNC Food Allergy Initiative, Department of Pediatrics, UNC School of Medicine, Chapel Hill, NC, United States

**Keywords:** food allergy, peanut allergy, oral tolerance, collaborative cross, airway sensitization

## Abstract

**Background:**

Early dietary introduction of peanut has shown efficacy in clinical trials and driven pediatric recommendations for early introduction of peanut to children with heightened allergy risk worldwide. Unfortunately, tolerance is not induced in every case, and a subset of patients are allergic prior to introduction. Here we assess peanut allergic sensitization and oral tolerance in genetically diverse mouse strains.

**Objective:**

We aimed to determine whether environmental adjuvant-driven airway sensitization and oral tolerance to peanut could be induced in various genetically diverse mouse strains.

**Methods:**

C57BL/6J and 12 Collaborative Cross (CC) mouse strains were fed regular chow or *ad libitum* peanut butter to induce tolerance. Tolerance was tested by attempting to sensitize mice via intratracheal exposure to peanut and lipopolysaccharide (LPS), followed by intraperitoneal peanut challenge. Peanut-specific immunoglobulins and peanut-induced anaphylaxis were assessed.

**Results:**

Without oral peanut feeding, most CC strains (11/12) and C57BL/6J induced peanut-specific IgE and IgG1 following airway exposure to peanut and LPS. With oral peanut feeding none of the CC strains nor C57BL/6J mice became sensitized to peanut or experienced anaphylaxis following peanut challenge.

**Conclusion:**

Allergic sensitization and oral tolerance to peanut can be achieved across a range of genetically diverse mice. Notably, the same strains that became allergic via airway sensitization were tolerized by feeding high doses of peanut butter before sensitization, suggesting that the order and route of peanut exposure are critical for determining the allergic fate.

## Introduction

The onset of peanut allergy typically occurs during childhood and is a potentially fatal disease. Unlike other common childhood food allergies such as egg or milk, peanut allergy is often lifelong (1–4). Allergic sensitization to peanut and other food allergens occurs due to a failure to initiate or maintain oral tolerance (5, 6). Children with atopic diseases such as eczema or asthma are at heightened risk for food allergies (7). Additionally, non-oral routes of peanut exposure, including cutaneous and airway exposure, have been linked to allergic sensitization in clinical studies and mouse models (6–9).

The potentially severe allergic responses to accidental exposure and lifelong persistence of peanut allergy have compelled research into means of preventing and treating peanut allergy (10–12). For prevention, the Learning Early About Peanut Allergy (LEAP) trial is a seminal study that found that early dietary introduction of peanut reduced rates of peanut allergy (13, 14). Subsequently, based on the observations in LEAP and other trials (15–17), the National Institutes of Allergy and Infectious Diseases (NIAID) and international experts updated recommendations for the early dietary introduction of peanut (18, 19). Follow-up studies generally reinforced the main finding from LEAP that early introduction of dietary peanut is efficacious, especially in higher-risk children (20). However, one recent study from Australia found that the overall rate of infant peanut allergy has not significantly decreased since introduction of the new feeding guidelines. Instead, early peanut introduction showed statistical benefits for infants with Australian ancestry but not those with East Asian ancestry (21). These findings suggest the existence of additional environmental and genetic confounders that limit the efficacy of early introduction of dietary peanut. A fuller understanding of these confounders may help guide peanut introduction recommendations and promote higher rates of oral tolerance.

Here, we aimed to determine whether environmental adjuvant-driven allergic sensitization and oral tolerance could be induced in genetically diverse mouse strains. For genetic diversity we surveyed C57BL/6J mice and 12 Collaborative Cross (CC) mouse strains. CC mice were specifically developed as a set of inbred mouse strains with genetically distinct backgrounds (22) and have been used to establish mouse models of human diseases (23). We chose to survey 12 CC strains because any set of greater than 10 CC strains is highly likely to sample each founder haplotype at least once at each locus, thus allowing an assessment of the impact that common genetic variation in the CC can have on a trait of interest. Each of the mouse strains were fed peanut butter or regular chow before intratracheal sensitization to peanut with the environmental adjuvant, lipopolysaccharide (LPS), and subsequently assessed for peanut allergy.

## Methods

### Mice

Mice from 12 Collaborative Cross (CC) mouse strains were obtained from the Systems Genetics Core Facility at UNC in November of 2022. The 12 strains included; CC001/Unc, CC004/TauUnc, CC006/TauUnc, CC012/GeniUncJ, CC013/GeniUncJ, CC015/UncJ, CC033/GeniUncJ, CC037/TauUnc, CC060/UncJ, CC061/GeniUncJ, CC068/TauUncJ, and CC071/TauUnc. C57BL/6J founding breeders were purchased from Jackson Laboratories (Bar Harbor, ME) and bred in-house. All mouse strains were maintained under specific pathogen-free conditions and raised on standard mouse chow 5V5R (Lab Diet subsidiary of Land O'Lakes Arden Hills, MN) which is free of peanut and soy allergen. Male CC (*n* = 3–4 per strain) and C57BL/6J mice (*n* = 8–10) between 5 and 14 weeks of age were used for experiments. The group sizes were based on power calculations from an earlier study (24) using male and female C57BL/6J mice and allowed for simultaneous screening of several CC strains. All animal experiments were approved by the Institutional Animal Care and Use Committee at the University of North Carolina at Chapel Hill.

### Reagents

Peanut protein extract was prepared from roasted de-fatted peanut flour (Golden Peanut, Alpharetta, GA) in PBS with 1 M NaCl as described previously (25). LPS from *Escherichia coli* 055:B5 was purchased from Sigma (St. Louis, MO). For peanut butter feeding mice were given Skippy P.B. Bites Double Peanut Butter (Hormel Foods, Austin, MN).

### Oral tolerance model

**Figure 1:**
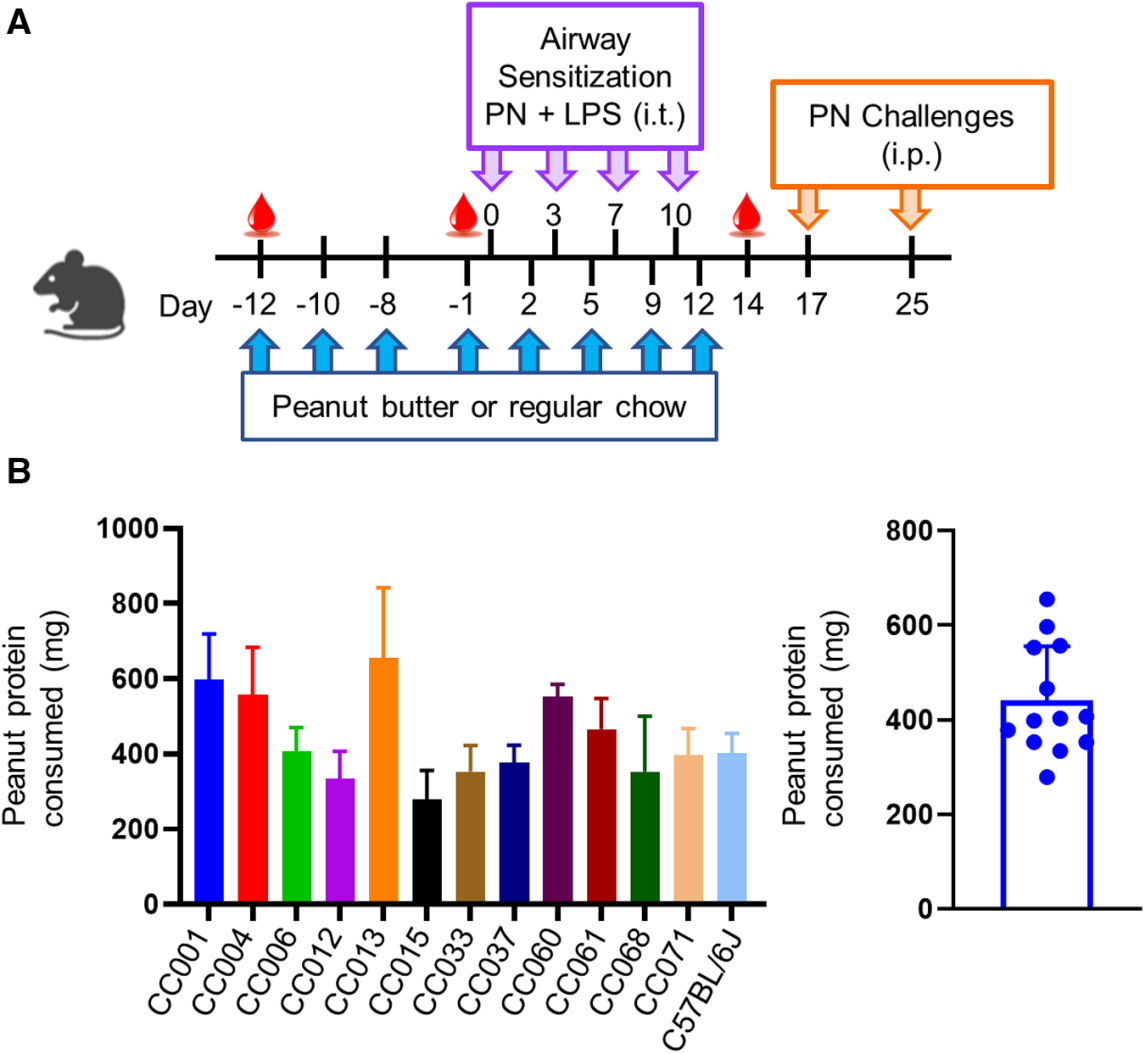
Model of peanut airway sensitization and peanut oral tolerance. (**A**) Experimental scheme showing peanut butter or regular chow feeding prior to airway sensitization with peanut (PN) and lipopolysaccharide (LPS) via intratracheal (i.t.) administration. Mice were challenged to peanut via intraperitoneal (i.p.) injection. (**B**) Average amount of peanut protein consumed per mouse per feeding (left), and per strain (right). Data are represented as means ± SD.

To test oral tolerance in CC strains, we modified a LEAP mouse model (26) as previously described (24). Briefly, mice were given peanut butter (PB) bites *ad libitum* for 24 h on days -12, -10, -8, and -1, before the sensitization protocol and on days 2, 5, 9, and 12, during sensitization, as shown in Figure 1A. Uneaten PB bites were collected and weighed to determine consumption. PB weight consumed was converted to peanut protein using the manufacturer reported protein content of 5 g per 28 g serving. Mice were sensitized by intratracheal (i.t.) administration with peanut and LPS twice weekly for two weeks. Briefly, mice were anesthetized with isoflurane and co-administered 150 ng peanut protein and 100 ng LPS in a total volume of 50 *μ*l as previously described (27, 28). Mice were bled on days -12, -1 and 14 to quantify peanut-specific IgE and IgG1. Mice were challenged by i.p. injection using 0.5 mg peanut protein on day 17 and 2.5 mg peanut protein on day 25 to help account for the variable susceptibility to i.p. peanut seen across mouse strains (23, 27). Core body temperatures were monitored every 15 min for one hour with a rectal thermometer (Physitemp, Clifton, NJ).

### Quantification of peanut-specific antibodies

Serum collected by submandibular bleed at the beginning of the model, before airway sensitization, and before peanut challenge (Figure 1A) was analyzed for peanut-specific IgE and IgG1 via ELISA, as described previously (27). Briefly, 96-well plates were coated with 20 µg/ml HSA-DNP (for standard curves) or peanut extract (for experimental samples) and blocked with 2% BSA in PBS-0.05% Tween. Samples were diluted 1:100 for peanut-specific IgE and 1:20,000 for peanut-specific IgG1 ELISAs. Standard curves ranging from 62.5–0.06 ng/ml for mouse IgE anti-DNP or from 2,000–2 ng/ml for mouse IgG1 anti-DNP (Accurate Chemicals, Westbury, NY) were generated via 1:2 serial dilutions. Plates were detected with HRP-goat anti-mouse IgE (1:10,000, Southern Biotech, Birmingham, AL) or HRP-goat anti-mouse IgG1 (1:40,000, Southern Biotech, Birmingham, AL). All plates were developed using TMB (Seracare, Milford, MA) and stopped using 2N sulfuric acid. Immunoglobulin ELISA plates were read at 450 nm using a microplate spectrophotometer (BioTek Instruments, Winooski, VT), and concentrations were calculated based on standard curves.

### Statistical analysis

GraphPad Prism version 9 was used to analyze all data. Paired t-tests were performed, and a *p*-value < 0.05 was considered significant.

## Results

### Varying quantities of peanut butter are consumed during *ad libitum* feeding

A modified oral tolerance model (24) was implemented, where mice were given peanut butter eight times before or during airway exposure to peanut plus LPS and assessed for sensitization and anaphylaxis to peanut as a readout of allergy (Figure 1A). Peanut consumption was monitored during *ad libitum* feeding; C57BL/6J and all CC strains consumed peanut, with an average consumption of ∼450 mg of peanut protein per feeding (Figure 1B). There was variation among strains that correlated with both age and weight, with average protein consumption ranging from ∼300–700 mg of peanut protein. No correlations were observed between amount of peanut butter eaten and the induction of peanut specific antibodies or anaphylaxis upon i.p. peanut challenge (data not shown).

### Peanut butter feeding prevents peanut allergy in genetically diverse mice

**Figure 2:**
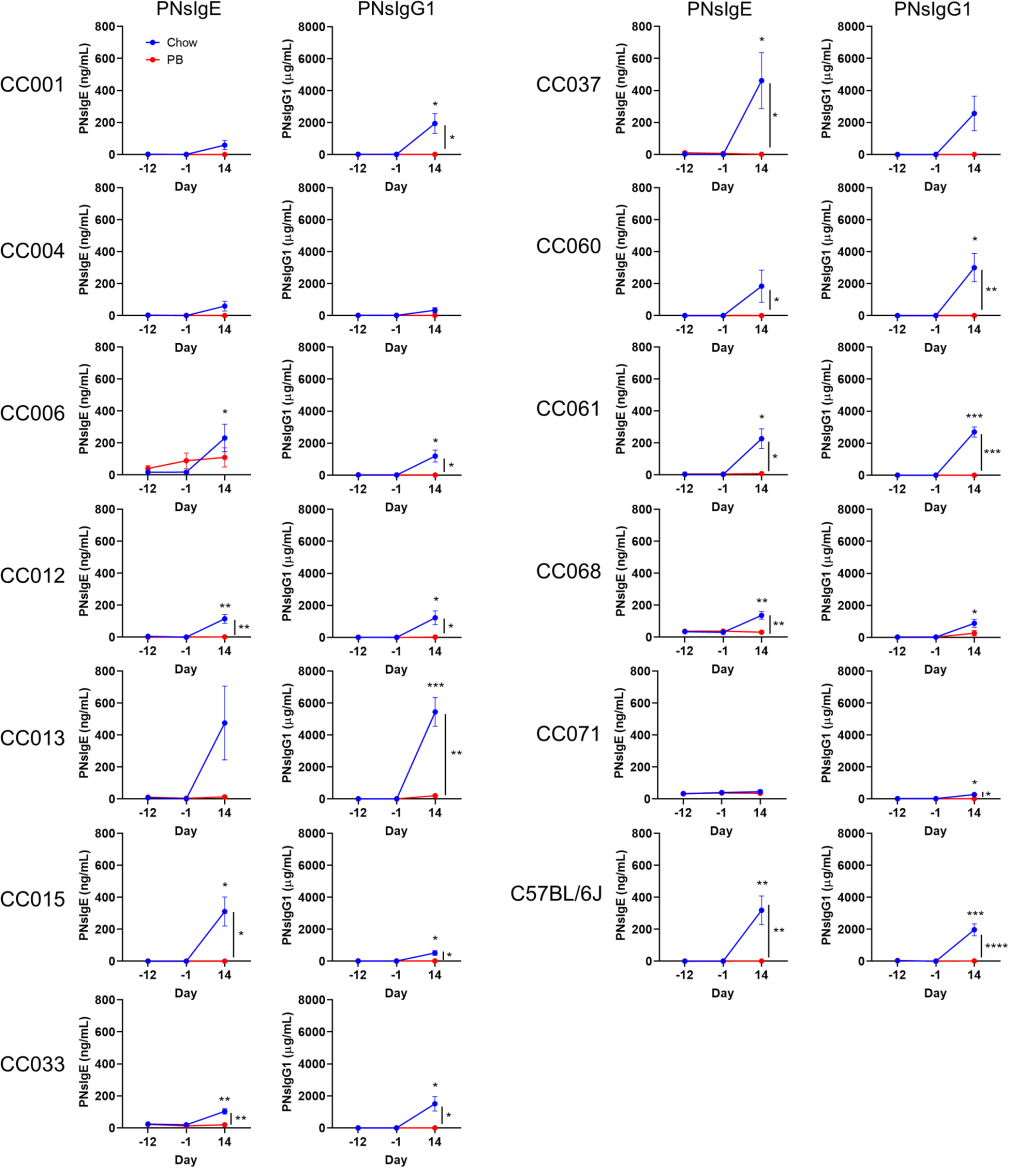
Serum peanut specific IgE and IgG1 throughout the experiment. Peanut specific IgE and IgG1 (respectively PNsIgE and PNsIgG1) from chow-fed (blue) or peanut butter (PB)-fed (red) mice on days -12, 1 and 14. Means ± SEM are shown. Statistical analysis was performed by Student's t-test; difference in chow-fed mice between day -12 and day 14 are indicated above the blue line, and difference between chow and PB-fed CC mice on day 14 are shown with a bracket to the right. No statistical differences were observed between day -12 and day -1 for chow- or PB-fed mice. *P < 0.05, **P < 0.01, ***P < 0.001.

Non-oral routes of peanut exposure, including skin and airway exposure, have been associated with peanut allergy (6, 13). In our model, airway exposure to peanut plus LPS was used as the sensitization regimen, and development of peanut-specific IgE and IgG1 were quantified. C57BL/6J mice and each of the CC strains except CC004/TauUnc totaling (11 out of 12) that received regular chow developed peanut-specific IgE and/or peanut-specific IgG1 after airway sensitization (Figure 2, blue lines; and Supplementary Tables S1, S2). Peanut-specific IgE was significantly higher after sensitization (day 14) compared to baseline (day -12) in C57BL/6J and 7 CC strains (Figure 2; left graphs, blue lines), and peanut-specific IgG1 was significantly higher after sensitization in C57BL/6J and 10 CC strains (Figure 2; right graphs, blue lines). In conclusion peanut-specific IgE and/or IgG1 can be induced by airway delivery of peanut plus LPS in several strains from a pool of genetically diverse mice.

Early oral exposure to food allergens is associated with immunological tolerance (5, 7, 16). In our oral tolerance model, the development of tolerance is inferred by comparing the responses of chow-fed mice to those of peanut butter-fed mice. Specifically, we assess if there is a reduced induction of peanut-specific IgE and/or IgG1. Of the 7 CC strains that produced statistically increased peanut-specific IgE after chow feeding, 6 had significantly lower peanut-specific IgE levels after peanut butter feeding (Figure 2; left graphs). Of the 10 CC strains that produced increased peanut-specific IgG1 in the normal chow fed group, 9 had significantly lower IgG1 in the peanut fed group (Figure 2; right graphs). The two strains that did not have statistically decreased peanut-specific antibodies, CC006/TauUnc for IgE and CC068/TauUncJ for IgG1, had peanut-specific antibodies that trended lower. Moreover, none of the peanut-fed mice produced statistically increased peanut-specific IgE or IgG1 after oral peanut feeding (day -1) or after the sensitization regimen (day 14) (Figure 2; red lines). In summary, dietary peanut prevented significant induction of peanut-specific IgE or IgG1 in each case where the corresponding chow-fed strains had elevated peanut-specific IgE and/or IgG1. These results demonstrate that peanut feeding promotes oral tolerance in genetically diverse mice.

**Figure 3:**
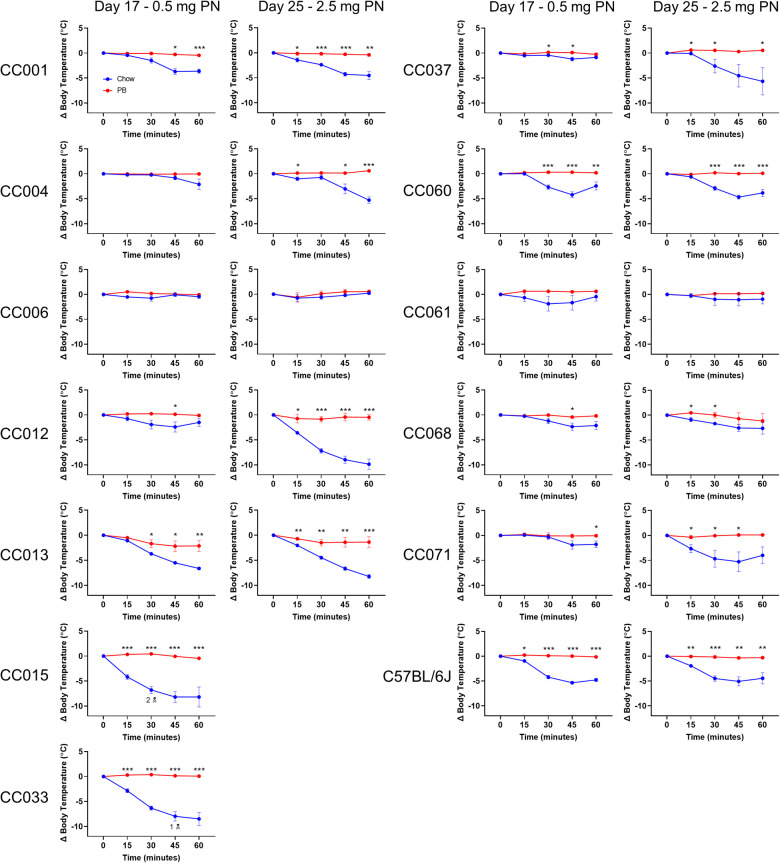
Peanut challenge results. Body temperature data in chow-fed (blue) and PB-fed (red) CC mice after the peanut (PN) challenge on days 17 and 25. Means ± SEM are shown. Statistical analysis was performed using Student's t-test, *P < 0.05, **P < 0.01, ***P < 0.001. The skull and crossbones symbol (*N*) indicates mouse death.

Peanut allergy was assessed by i.p. peanut challenge administered first on day 17, with 0.5 mg peanut protein. CC015/UncJ and CC033/GeniUncJ mice fed regular chow had severe and even fatal reactions following peanut challenge. In contrast, most of the other strains did not experience anaphylaxis (defined as a greater than 3°C temperature decrease) (Figure 3; left graphs) following a 0.5 mg peanut protein challenge. To investigate whether this was related to the dose of peanut given during the challenge, mice were rechallenged with a five-fold higher dose of peanut protein (2.5 mg) on day 25. In the second challenge, most CC strains (9 of 12) fed regular chow experienced anaphylaxis (Figure 3; right graphs); however, CC006/TauUnc, CC061/GeniUncJ, and CC068/TauUncJ did not react even at the higher dose. In contrast, when the 9 reactive CC strains were fed peanut butter before the sensitization regimen, none reacted, indicating oral tolerance induction.

## Discussion

Few mouse models of airway sensitization to peanut exist (26–29). Our group and others have demonstrated airway sensitization to peanut in the conventional mouse strains C57BL/6J and BALB/cJ (26–29). While these demonstrate a proof of concept that airway sensitization to peanut can occur, it is unclear whether this happens in mice from other genetic backgrounds. Our data demonstrates that 11 of the 12 CC strains screened developed peanut-specific IgE and/or IgG1 following airway exposure to a low dose of peanut (150 ng) and the environmental adjuvant LPS. The induced peanut-specific IgE and/or IgG1 in C57BL/6J and 9 of the 12 CC strains additionally correlated with a change in body temperature during the anaphylaxis challenge (Supplementary Figure S1). These results suggest that the airway route of sensitization is plausible across genetic backgrounds and not strain or model specific. These findings also provide credibility for the airway as a route of sensitization in humans.

Oral tolerance induction in mice has been shown for various antigens, including food proteins (30–33). Typically, these models are conducted in conventional mouse strains such as C57BL/6 and BALB/c, but some studies have been performed with the model allergen ovalbumin in genetically diverse strains of mice (34). To our knowledge, oral tolerance to peanut has not been investigated in the CC or other mouse genetic reference panels. Here, we investigated oral tolerance induction to peanut in C57BL/6J mice and 12 CC strains by feeding peanut butter before an airway sensitization regimen. None of the CC strains fed peanut butter reacted during the peanut challenge, but 9 of the 12 CC strains experienced anaphylaxis when fed regular chow. These results demonstrate that oral tolerance to peanut is induced in various genetically distinct mouse strains, which more closely resemble the genetically outbred human population.

Two strains (CC015/UncJ and CC033/GeniUncJ) had severe anaphylaxis following a 0.5 mg peanut protein challenge, while three strains (CC006/TauUnc, CC061/GeniUncJ and CC068/TauUncJ) did not experience anaphylaxis. Interestingly, each of these five strains generate peanut-specific IgE and IgG1, suggesting that their heightened or lacking reactivity to peanut may be due to upstream differences including peanut-specific antibody affinity (35), or mast cell or basophil number or reactivity (23, 36, 37). The non-reacting strains may have lower affinity antibodies or a higher threshold for peanut and might react if given a higher dose during the challenge. On the other hand, the severely reacting strains may have higher affinity antibodies or a lower threshold for peanut and more reactive mast cells or basophils. High affinity peanut-specific antibodies or highly reactive mast cells or basophils may also explain the anaphylaxis observed in CC071/TauUnc and CC004/TauUnc strains of mice, which made very low levels of peanut-specific IgE and IgG1, yet reacted to peanut. The varying responses across all 12 CC strains demonstrate the impact of genetic determinants on both oral tolerance and sensitization to peanut. In human studies, several candidate genes have been associated with the development of food allergy, including FLG, HLA, and MALT1 (38, 39). Additionally, there are environmental factors that confound the risk of food allergy, including levels of air pollution, microbial diversity, and residential greenness (40). Future work needs to be done to identify specific genetic factors and elements of the external exposome associated with oral tolerance and peanut allergy.

Further mechanistic studies should be performed to gain a greater insight into genetic and environmental factors that influence peanut allergy. Specifically, genetic risk factors can be investigated by quantitative trait locus mapping through cross-breeding of CC strains and analysis of allergy endpoints (41). Additionally, differences in initiating immune responses including dendritic cell activation and migration, and cytokine production by innate lymphoid cells can be monitored by flow cytometry or RNAseq. Differences in the adaptive response phase can be monitored by *ex vivo* peanut restimulation of cells from draining lymph nodes. Finally, differences in antibody production and affinity could be contributing to dose reactivity thresholds and could be measured in future experiments. Effects of exposome exposure can similarly be determined by varying the environmental adjuvant used during the sensitization phase of our mouse model. Any newly discovered genetic risk factors could potentially be exploited as diagnostic tools to screen for food allergy before dietary allergen introduction in infants. Similarly, pediatricians could inform parents about environmental factors that may have a detrimental or beneficial effect on allergy onset.

Given that the majority of CC strains screened can become tolerant or allergic depending on the initial peanut exposure, it is clear that the order and route of peanut exposure are critical for distinguishing oral tolerance and sensitization. Regardless of genetic background, all CC strains fed peanut butter were resistant to peanut-induced anaphylaxis. These results further emphasize findings from the LEAP study that demonstrate the importance of early introduction of peanut for preventing allergy.

## Data Availability

The raw data supporting the conclusions of this article will be made available by the authors, without undue reservation.
