# Supplementary material for: Peanut butter feeding induces oral tolerance in genetically diverse collaborative cross mice
Source: Front Allergy. 2023 Jul 17;4:1219268. doi: 10.3389/falgy.2023.1219268 (PMC10387557; doi:10.3389/falgy.2023.1219268)
Supplement: Supplementary file 1 [file Table1.docx]

**Supplemental Table 1. Serum peanut-specific IgE**

| **Peanut- specific IgE**  **ng/mL** |  | Day -12 | Day -1 | Day 14 | p-value  Day -12 v. Day 14 | p-value Day 14 Chow v. PB |
| --- | --- | --- | --- | --- | --- | --- |
|  | N | Mean ± SEM | Mean ± SEM | Mean ± SEM |  |  |
| CC001 Chow | 4 | 1.5 ± 1.0 | 0.7 ± 0.7 | 58.8 ± 28.7 | 0.093 | 0.089 |
| CC004 Chow | 4 | 2.7 ± 1.6 | 0.0 ± 0.0 | 59.8 ± 30.5 | 0.111 | 0.100 |
| CC006 Chow | 4 | 16.9 ± 7.7 | 17.9 ± 5.8 | 231.1 ± 86.2 | **0.048** | 0.292 |
| CC012 Chow | 4 | 5.0 ± 5.0 | 0.0 ± 0.0 | 114.8 ± 27.5 | **0.008** | **0.006** |
| CC013 Chow | 4 | 7.4 ± 4.7 | 1.3 ± 1.3 | 475.5 ± 230.8 | 0.089 | 0.092 |
| CC015 Chow | 4 | 0.0 ± 0.0 | 0.0 ± 0.0 | 311.0 ± 91.0 | **0.014** | **0.014** |
| CC033 Chow | 4 | 24.5 ± 4.6 | 19.9 ± 2.8 | 103.9 ± 16.7 | **0.004** | **0.003** |
| CC037 Chow | 4 | 2.5 ± 1.4 | 1.1 ± 1.1 | 461.9 ± 175.0 | **0.039** | **0.039** |
| CC060 Chow | 3 | 0.0 ± 0.0 | 0.0 ± 0.0 | 184.1 ± 100.7 | 0.142 | **0.046** |
| CC061 Chow | 4 | 3.6 ± 2.2 | 2.8 ± 1.6 | 227.1 ± 62.0 | **0.011** | **0.031** |
| CC068 Chow | 4 | 34.3 ± 3.1 | 30.3 ± 1.7 | 136.4 ± 24.0 | **0.006** | **0.006** |
| CC071 Chow | 4 | 32.9 ± 1.8 | 40.6 ± 6.4 | 45.8 ± 10.8 | 0.283 | 0.410 |
| C57BL/6J Chow | 8 | 0.0 ± 0.0 | 0.9 ± 0.7 | 318.7 ± 89.8 | **0.003** | **0.001** |
| CC001 PB | 4 | 1.5 ± 1.1 | 0.0 ± 0.0 | 0.5 ± 0.4 | 0.438 |  |
| CC004 PB | 4 | 1.9 ± 1.4 | 1.9 ± 0.9 | 0.5 ± 0.4 | 0.376 |  |
| CC006 PB | 4 | 40.2 ± 17.8 | 88.8 ± 48.3 | 109.8 ± 60.2 | 0.310 |  |
| CC012 PB | 4 | 0.0 ± 0.0 | 0.0 ± 0.0 | 1.6 ± 1.6 | 0.356 |  |
| CC013 PB | 4 | 10.7 ± 7.9 | 4.6 ± 2.7 | 13.0 ± 4.9 | 0.812 |  |
| CC015 PB | 4 | 0.0 ± 0.0 | 0.0 ± 0.0 | 0.0 ± 0.0 | N/A |  |
| CC033 PB | 4 | 22.3 ± 2.8 | 12.9 ± 4.3 | 19.6 ± 3.2 | 0.556 |  |
| CC037 PB | 4 | 11.1 ± 9.9 | 6.7 ± 5.6 | 2.0 ± 1.2 | 0.396 |  |
| CC060 PB | 5 | 0.0 ± 0.0 | 0.0 ± 0.0 | 0.0 ± 0.0 | N/A |  |
| CC061 PB | 3 | 6.0 ± 2.0 | 5.5 ± 2.4 | 8.5 ± 2.3 | 0.349 |  |
| CC068 PB | 4 | 36.8 ± 2.1 | 37.5 ± 4.6 | 30.8 ± 8.4 | 0.517 |  |
| CC071 PB | 4 | 34.0 ± 2.1 | 38.0 ± 4.9 | 35.5 ± 4.5 | 0.768 |  |
| C57BL/6J PB | 10 | 0.0 ± 0.0 | 0.0 ± 0.0 | 0.7 ± 0.7 | 0.331 |  |
