# Supplementary material for: Peanut butter feeding induces oral tolerance in genetically diverse collaborative cross mice
Source: Front Allergy. 2023 Jul 17;4:1219268. doi: 10.3389/falgy.2023.1219268 (PMC10387557; doi:10.3389/falgy.2023.1219268)
Supplement: Supplementary file 2 [file Table2.docx]

**Supplemental Table 2. Serum peanut-specific IgG1**

| **Peanut- specific IgG1**  **µg/mL** |  | Day -12 | Day -1 | Day 14 | p-value  Day -12 v. Day 14 | p-value Day 14 Chow v. PB |
| --- | --- | --- | --- | --- | --- | --- |
|  | N | Mean ± SEM | Mean ± SEM | Mean ± SEM |  |  |
| CC001 Chow | 4 | 20.1 ± 3.8 | 16.7 ± 0.6 | 1,949.7 ± 618.8 | **0.021** | **0.020** |
| CC004 Chow | 4 | 17.2 ± 1.1 | 15.9 ± 0.9 | 339.4 ± 160.1 | 0.091 | 0.091 |
| CC006 Chow | 4 | 22.3 ± 4.1 | 17.4 ± 1.7 | 1,198.6 ± 370.2 | **0.019** | **0.019** |
| CC012 Chow | 4 | 8.5 ± 0.4 | 7.2 ± 0.4 | 1,233.0 ± 427.2 | **0.029** | **0.030** |
| CC013 Chow | 4 | 8.0 ± 0.6 | 8.1 ± 0.7 | 5,448.7 ± 903.6 | **<0.001** | **0.001** |
| CC015 Chow | 4 | 11.4 ± 0.9 | 10.2 ± 0.3 | 514.3 ± 149.8 | **0.015** | **0.016** |
| CC033 Chow | 4 | 0.0 ± 0.0 | 0.0 ± 0.0 | 1,507.7 ± 453.2 | **0.016** | **0.016** |
| CC037 Chow | 4 | 0.0 ± 0.0 | 0.0 ± 0.0 | 2,569.4 ± 1,076.8 | 0.054 | 0.054 |
| CC060 Chow | 3 | 0.0 ± 0.0 | 0.0 ± 0.0 | 3,004.0 ± 883.8 | **0.027** | **0.003** |
| CC061 Chow | 4 | 0.0 ± 0.0 | 0.0 ± 0.0 | 2,700.0 ± 319.7 | **<0.001** | **<0.001** |
| CC068 Chow | 4 | 21.3 ± 0.4 | 23.1 ± 1.2 | 881.9 ± 247.6 | **0.013** | 0.084 |
| CC071 Chow | 4 | 21.7 ± 1.0 | 23.4 ± 1.1 | 276.3 ± 74.0 | **0.014** | **0.013** |
| C57BL/6J Chow | 8 | 35.5 ± 10.4 | 5.1 ± 1.8 | 1,967.5 ± 378.3 | **<0.001** | **<0.001** |
| CC001 PB | 4 | 19.8 ± 2.0 | 15.6 ± 1.4 | 16.8 ± 1.1 | 0.229 |  |
| CC004 PB | 4 | 18.8 ± 1.8 | 17.9 ± 0.7 | 17.7 ± 1.1 | 0.616 |  |
| CC006 PB | 4 | 20.6 ± 1.2 | 18.1 ± 1.2 | 18.1 ± 0.9 | 0.148 |  |
| CC012 PB | 4 | 8.1 ± 0.5 | 5.0 ± 0.7 | 25.0 ± 8.6 | 0.097 |  |
| CC013 PB | 4 | 11.0 ± 1.1 | 9.5 ± 1.4 | 201.2 ± 90.8 | 0.081 |  |
| CC015 PB | 4 | 14.0 ± 0.5 | 14.1 ± 1.0 | 14.4 ± 0.4 | 0.582 |  |
| CC033 PB | 4 | 0.0 ± 0.0 | 0.0 ± 0.0 | 0.0 ± 0.0 | N/A |  |
| CC037 PB | 4 | 0.0 ± 0.0 | 0.0 ± 0.0 | 0.0 ± 0.0 | N/A |  |
| CC060 PB | 5 | 0.0 ± 0.0 | 0.0 ± 0.0 | 0.0 ± 0.0 | N/A |  |
| CC061 PB | 3 | 0.0 ± 0.0 | 0.0 ± 0.0 | 0.0 ± 0.0 | N/A |  |
| CC068 PB | 4 | 24.3 ± 1.9 | 24.3 ± 2.6 | 263.9 ± 167.3 | 0.202 |  |
| CC071 PB | 4 | 19.1 ± 0.6 | 19.3 ± 0.4 | 18.8 ± 0.9 | 0.731 |  |
| C57BL/6J PB | 10 | 30.8 ± 11.2 | 4.1 ± 1.7 | 16.2 ± 8.1 | 0.307 |  |
