# Supplementary material for: Peanut butter feeding induces oral tolerance in genetically diverse collaborative cross mice
Source: Front Allergy. 2023 Jul 17;4:1219268. doi: 10.3389/falgy.2023.1219268 (PMC10387557; doi:10.3389/falgy.2023.1219268)
Supplement: Supplementary file 3 [file Datasheet1.docx]

*Supplementary Material*

**Results**

*IgE and/or IgG1 correlate with anaphylaxis in various CC strains*

Serum concentrations of allergen specific IgE typically correlate with food allergy in humans. In mouse models, anaphylaxis may be IgE and/or IgG dependent (1). To explore if anaphylaxis in our model correlated with serum concentrations of peanut-specific IgE and/or IgG1 we plotted serum antibody concentrations against the observed body temperature drop during the 0.5 mg peanut protein challenge. Both IgE and IgG1 antibodies were correlated with anaphylaxis in 3 CC strains (CC004/TauUnc, CC033/GeniUncJ, and CC068/TauUncJ) and C57BL/6J, peanut-specific IgE but not IgG1 correlated with anaphylaxis in 3 CC strains (CC006/TauUnc, CC012/GeniUncJ, CC061/GeniUncJ), peanut-specific IgG1 but not IgE correlated with anaphylaxis in 3 CC strains (CC013/GeniUncJ, CC015/UncJ, CC060/UncJ), and 3 CC strains showed no correlation between peanut-specific antibodies and anaphylaxis (CC001/Unc, CC037/TauUnc, CC071/TauUnc) (**Supplemental Figure 1**). In cases where a statistical correlation was not found peanut-specific IgE and IgG1 nonetheless tended to increase with body temperature drop except for in strains CC071/TauUnc and CC006/TauUnc. Additional studies varying the sensitization procedure or blocking IgG dependent anaphylaxis would help to further elucidate difference in IgE or IgG1 dependent anaphylaxis between CC strains. A mouse model of peanut allergy that does not display IgG dependent anaphylaxis would be of interest because it is likely to better mimic human disease.

**References**

1. Dolence JJ, Kobayashi T, Iijima K, Krempski J, Drake LY, Dent AL, et al. Airway Exposure Initiates Peanut Allergy by Involving the Il-1 Pathway and T Follicular Helper Cells in Mice. *J Allergy Clin Immunol* (2018) 142(4):1144-58 e8. Epub 20171214. doi: 10.1016/j.jaci.2017.11.020.

**Supplemental Figure 1. Correlation plots comparing peanut-specific IgE or IgG1 with body temperature drop.** Peanut-specific IgE or IgG1 (PNsIgE and PNsIgG1, respectively) on day 14 from individual mice is plotted against the corresponding temperature change at 45 minutes following the day 17 challenge with 0.5 mg peanut protein. Due to mouse deaths at later timepoints body temperature change at 15 minutes and 30 minutes are shown for strains CC015/UncJ and CC033/GeniUncJ, respectively instead of 45 minute. The data are fit with a simple linear regression and the plots include the R^2^ and p-values, as well as the best fit line (solid) and 95% confidence interval (dotted).
