# Supplementary figures and images for: Peanut butter feeding induces oral tolerance in genetically diverse collaborative cross mice
Source: Front Allergy. 2023 Jul 17;4:1219268. doi: 10.3389/falgy.2023.1219268 (PMC10387557; doi:10.3389/falgy.2023.1219268)

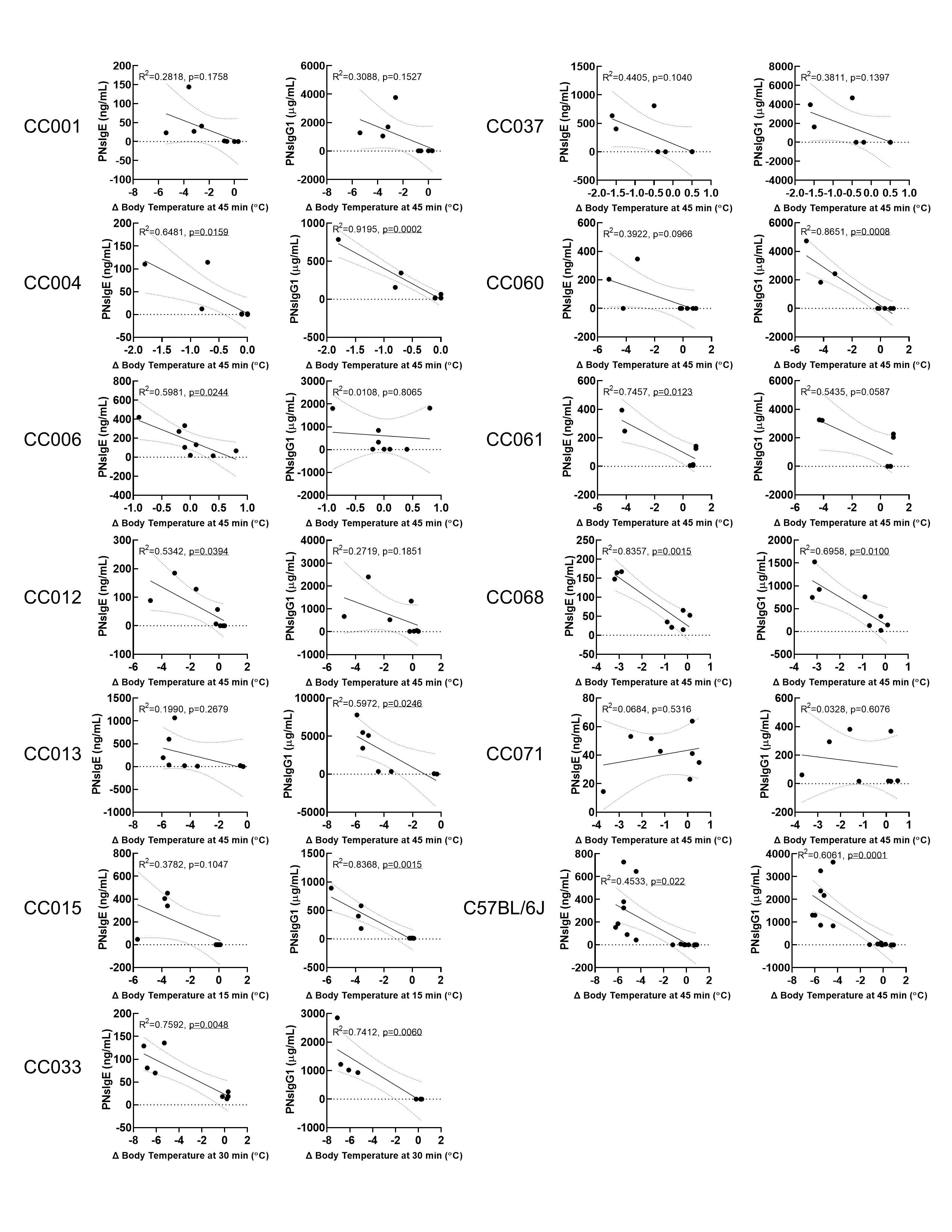

Supplement: Supplementary file 4 [file Image1.tif]
